# Effects of β2 Integrins on Osteoclasts, Macrophages, Chondrocytes, and Synovial Fibroblasts in Osteoarthritis

**DOI:** 10.3390/biom12111653

**Published:** 2022-11-08

**Authors:** Tiantian Hu, Zhan Zhang, Chunbo Deng, Xun Ma, Xueyong Liu

**Affiliations:** 1 Department of Rehabilitation, Shengjing Hospital of China Medical University, Shenyang 110134, China; 2 Department of Orthopaedics, Shengjing Hospital of China Medical University, Shenyang 110134, China; 3 Department of Orthopaedics, Fengtian Hospital of Shenyang Medical College, Shenyang 110134, China

**Keywords:** osteoarthritis, integrins, osteoclasts, macrophages, chondrocytes, fibroblasts

## Abstract

β2 integrins are transmembrane receptors that exist widely in human immune cells and participate in pathological processes such as chronic inflammation, thrombosis, and malignant tumor formation. They mainly mediate intercellular adhesion, coordinate the ingestion of extracellular matrix components, and regulate cytoskeleton formation, thereby regulating cell signaling. Osteoarthritis (OA) is a chronic joint disease that causes joint pain and increases disease burden; it has a high prevalence among populations worldwide. Previous studies have reported that β2 integrins are overexpressed in OA and may play an essential role in the occurrence of OA. The important roles of β2 integrins in the maturation and differentiation of osteoclasts, the regulation of bone homeostasis, and the polarization and migration of macrophages have also been reported. The present review aims to highlight the role of β2 integrins in OA pathogenesis and outline their potential for serving as therapeutic targets.

## 1. Introduction

Osteoarthritis (OA) is a chronic degenerative joint disease that may induce cartilage denaturation and destruction, osteophyte formation, and synovitis development [1]. Recent research has found that innate immunity plays a vital role in the occurrence of OA [2]. Inflammatory cells formed by macrophages, T and B lymphocytes, and other leukocytes infiltrate the synovial tissue in OA and release various types of cytokines and chemokines, including interleukin-1 beta (IL-1β), IL-6, tumor necrosis factor-alpha (TNF-α), and nerve growth factor (NGF) [3]. Additionally, immune microenvironment imbalance caused by macrophage polarization has influences on OA pathogenesis. At present, OA is believed to be the consequence of interactions between low-grade systemic inflammation and joint-related tissue degeneration [4].

Integrins are heterodimeric transmembrane receptors composed of α and β subunits. Widely present in animal and plant cells, they mediate intercellular and extracellular matrix (ECM) interactions and participate in physiological processes such as hemostasis, wound healing, immunity, and developmental biology [5,6]. Humans possess a total of 18 α subunits and 8 β subunits, from which 24 different integrin heterodimers with a high degree of tissue specificity are generated through noncovalent bonding [7]. Multiple studies have demonstrated that β1 and β3 integrins can contribute to mediating cell adhesion in chondrocyte mechanotransduction and regulating cell proliferation, differentiation, and cartilage homeostasis in OA pathogenesis via the ligand–receptor interactions between integrins and the ECM proteins [8,9]. β2 integrins, specifically expressed on leukocyte surfaces, play a pivotal role in the processes of multiple inflammatory diseases, including systemic lupus erythematosus and rheumatoid arthritis (RA) [10,11].

Although the relationships between integrin dysfunction involving β1 and β3 integrins and the pathogenesis of OA have been previously reported [12], whether β2 integrins also serve as important factors in OA pathogenesis is not fully elucidated yet. Moreover, the further roles of β2 integrins in the maturation and differentiation of osteoclasts (OCs), the polarization and migration of macrophages, and the maintenance of bone homeostasis have also been reported in recent years. Furthermore, several studies have found that the expression levels of β2 integrins were upregulated in cartilage, synovial and adipose tissues under the pathological conditions of OA [13,14]. Therefore, this review focuses on the functional roles of β2 integrins in OA progression, including cell events, especially in osteoclasts, macrophages, chondrocytes, and synovial fibroblasts. A clear understanding of the role of β2 integrins in OA may contribute to a deep understanding of pathogenesis and the potential considerations of therapeutic approaches.

## 2. Overview of β2 Integrins

**Figure 1:**
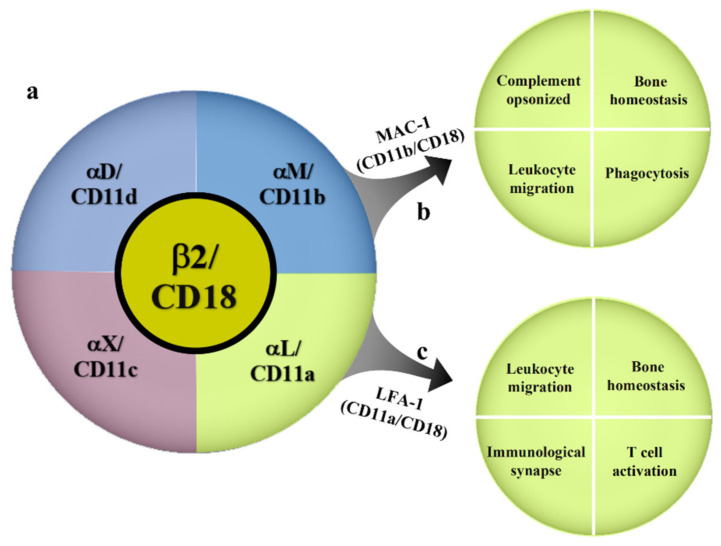
Classification of β2 integrins and the major cellular functions of LFA-1 and Mac-1. a. β2 integrins exist as a combination of a β2/CD18 subunit and four different α subunits. b. Mac-1 consists of αM/CD11b and β2/CD18. It modulates the complement-opsonized bone homeostasis, leukocyte migration, and phagocytosis. c. LFA-1 consists of αL/CD11a and β2/CD18. It regulates the leukocyte migration, bone homeostasis, immunological synapse, and T-cell activation.

β2 integrins are heterodimeric surface receptors formed from the respective combination of a β2 subunit (CD18) encoded by the integrin subunit beta 2 (*ITGB2*) gene and four different α subunits (αM, αL, αD, αX) (Figure 1). These include αLβ2 (leukocyte function-associated antigen-1, LFA-1, also known as CD11a/CD18), αMβ2 (macrophage differentiation antigen-1, Mac-1, also known as CD11b/CD18), αXβ2a (CD11c/CD18), and αDβ2 (CD11d/CD18) [15]. Two subunits undergo intracellular dimerization to form a complete β2 integrin, which is subsequently integrated into the cell membrane. Therefore, individual subunits cannot be detected on cell surfaces [16]. β2 integrins mainly mediate the adhesion of leukocytes to vascular endothelium, causing them to migrate and roll within blood vessels and ultimately leave the vasculature for recruitment into inflamed or injured tissue. The existing literature on OA pathogenesis has mainly focused on LFA-1 and Mac-1, whereas studies on the relationships of the CD11c/CD18 and CD11d/CD18 integrins with OA are relatively scarce. LFA-1 is expressed on all leukocytes and is also the only β2 integrin expressed on T cells [17]. Previous research has found that LFA-1 mainly binds six types of ligands, with the binding affinity to intercellular adhesion molecule-1 (ICAM-1) being the highest [18,19]. LFA-1 plays a crucial role in immune processes such as leukocyte migration and activation, immune synapse formation, and the regulation of T-cell differentiation [20,21,22,23]. Mac-1 differs from LFA-1 in that it is mainly expressed on myeloid cells, such as polymorphonuclear neutrophils (PMNs), monocytes/macrophages, dendritic cells (DCs), and hematopoietic stem cell-derived OC precursors (pre-OCs). It is also regarded as a representative cell surface receptor of macrophages [24]. Mac-1 is capable of binding complement component 3 (C3) with high affinity and participating in the opsonization and removal of pathogens, tumor cells, and immune complexes. Therefore, it is also known as complement receptor 3 (CR3) [25,26]. Mac-1 also participates in biological processes such as ECM degradation and remodeling, immune tolerance [27], and the delay of neutrophil apoptosis.

**Figure 2:**
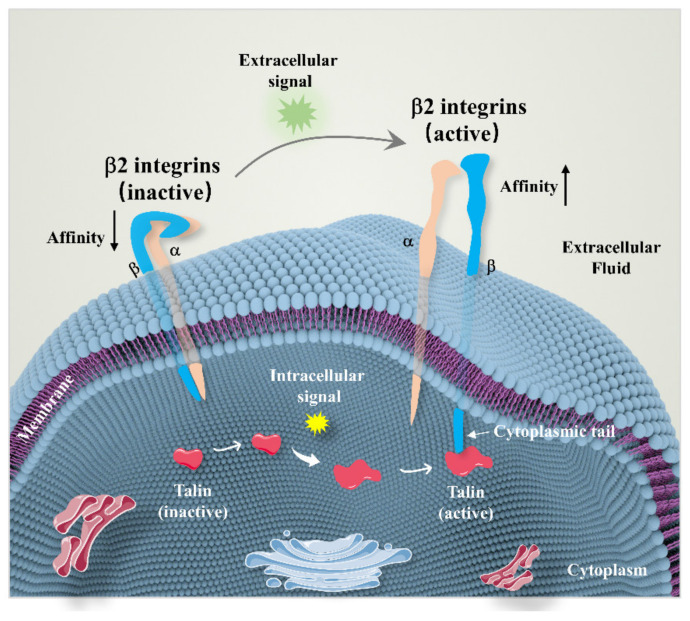
The affinity of β2 integrins can be regulated by bonding with talin. Talin is a large focal adhesion protein that interacts with the intracellular domains of β2 integrins (cytoplasmic tail). The interaction between talin and β2 integrins can enhance the affinity of integrins and ECM proteins via activating the β2 integrins.

β2 integrin consists of extracellular domain, cysteine stalk, and cytosolic tail. The cytosolic tail interacts with intracellular signaling proteins such as focal adhesion kinase (FAK), talin, paxillin, and vinculin to mediate the signaling transduction and regulate cell function [28]. Under normal conditions, β2 integrins are expressed in a low-affinity state on cell surfaces, that is, the extracellular domain is in a bent–closed state and the presence of leukocytes in blood vessels does not induce an inflammatory response [29]. Affinity for β2 integrin ligands is dependent on the transfer of allosteric factors between the α and β subunits [16]. Talin is a large focal adhesion protein that interacts with the intracellular domains of β2 integrins [30]. Studies have found that the binding of talin to the cytoplasmic tails of β subunits triggers the separation of α and β subunit cytoplasm from the transmembrane domain. When α and β subunit cytoplasmic tails bind to the salt bridge and ligands bind to the binding sites, the extracellular domain changes from a bent conformation to a highly extended conformation, thereby enhancing the binding affinity of integrins to ligands [31,32] (Figure 2). Cells maintain dynamic cell–ECM contact by modulating cytoskeleton filaments and their connections with transmembrane proteins [33]. The cytoskeleton, a key cellular component, is composed of a system of filaments that includes microtubules, microfilaments, and intermediate filaments. It forms the basis for the maintenance of cell morphology and the transmission of transmembrane signals [34]. Integrins can probe the physical features of the microenvironment and adjust their own tensional state through actomyosin contractility and the organization of the F-actin cytoskeleton to counterbalance extracellular forces, which launch the mechanotransduction process. The binding of high-affinity β2 integrins to specific ligands may result in cytoskeleton rearrangement, thereby mediating cellular signaling from outside to inside [33,35].

## 3. Role of β2 Integrins in OA Pathogenesis

Previous studies have found that genes encoding β2 integrins are significantly upregulated in OA, which leads to the conjecture that β2 integrins play a key role in the occurrence of OA. Hopwood et al. conducted a microarray analysis of gene expression profiles of OA and control bone samples, and found that a subset of genes involved in OC function, including *ITGB2*, which encodes for the β subunit of β2 integrins, were differentially expressed in OA bone [36]. In a similar study, Fan et al. performed an integrated analysis of gene expression in OA. The expression of the candidate gene *ITGB2* was markedly upregulated in OA compared with that of normal tissue, and pathway analysis revealed that osteoclastogenesis may be related to OA [37]. Another gene expression analysis conducted by Sun et al. revealed that *ITGB2* expression was remarkably upregulated in OA meniscal cells compared with that in normal meniscal cells and closely associated with the pathway of phosphate metabolism [38].

β2 integrins have previously been demonstrated to be strongly expressed in synovial fluid and the synovial tissue of inflammatory arthritis, including OA, RA, juvenile idiopathic arthritis (JIA), and spondyloarthritis (SpA) [39,40]. Related studies, although scarce, have indicated that the upregulation of β2 integrins also exists in the synovial tissue of osteoarthritic mice with hyperlipidemia. Compared with control mice, osteoarthritic mice with hyperlipidemia had an increased expression of CD11c(+) F4/80(+) CD11b(+) macrophages in synovial and adipose tissues [13,41]. The leukocyte infiltration of synovial fluid and synovial tissue indicates inflammatory arthritis, and leukocyte recruitment is an important element in the induction of the arthritic state. Studies have found that almost all types of β2 integrins are capable of mediating leukocyte migration and activation. Using CD18 null mice and blocking monoclonal antibodies (mAbs), Watts et al. demonstrated that LFA-1 expression is essential for the induction of arthritis. Blocking mAbs further revealed an ongoing requirement for the adhesive function of LFA-1 in disease perpetuation [42]. Such evidence suggests that β2 integrins may have a pro-inflammatory role in OA.

### 3.1. Effects of β2 Integrins on OCs

OCs are the only cells responsible for the resorption of bone, and abnormal subchondral bone remodeling is an important part of the occurrence and development process of OA [43]. Imaging examinations have revealed the presence of changes in the subchondral bone microenvironment in OA-affected joints, including early bone loss and late osteosclerosis. The dynamic equilibrium maintained between OC-induced bone destruction and osteoblast (OB)-mediated bone formation within a normal human body is known as bone homeostasis. In recent years, mounting evidence has demonstrated that abnormal OC differentiation and maturation upsets bone homeostasis and triggers abnormal subchondral bone loss in the early stages of OA [44,45]. The pathogenesis of OA is also related to chondrocyte apoptosis and ECM degradation in articular cartilage, with OCs serving a role in these pathogenic processes. A study that investigated the effects of blocking OC-related receptors found that the inhibition of OC differentiation attenuated chondrocyte apoptosis and cartilage matrix destruction in OA [46]. OCs can be formed from pre-OCs under stimulation by the macrophage colony-stimulating factor (M-CSF) and receptor activator of nuclear factor-kappa B ligand (RANKL), which are both expressed by OBs. RANKL on the surface of OBs can interact with RANK receptors on that of pre-OCs under the stimulation of M-CSF, which mediates the differentiation of pre-OCs into activated OCs [47]. Recent studies have found that β2 integrins also participate in osteoclastogenesis.

ICAM-1 is expressed on OB surfaces in OA patients, and the expression of LFA-1 is restricted to the surfaces of pre-OCs. The intercellular interactions of LFA-1/ICAM-1 exert certain effects on the maintenance of bone homeostasis [48,49]. Tanaka et al. used anti-LFA-1 antibodies to block LFA-1/ICAM-1 adhesion between OBs and pre-OCs, which reduced the production of the osteoclast differentiation factor (ODF) and consequently decreased the formation of OC-like multinucleated cells. Therefore, it can be deduced that high-affinity LFA-1/ICAM-1 adhesion is a prerequisite for the efficient function of membrane-bound ODF during OC maturation [50]. Further research revealed that the LFA-1/ICAM-1 axis may exert synergistic effects on OC formation by participating in the upregulation of RANKL expression [51]. Vinculin, which is a cytoplasmic actin-binding protein, plays an important role in cell–cell and cell–matrix adhesions [52]. Under the effects of LFA-1/ICAM-1 interactions, vinculin and β2 integrins are recruited to the peripheral regions of the actin core of OC podosomes, thereby promoting podosome formation in OCs [53,54]. An in vitro experiment that examined the blockade of LFA-1/ICAM-1 binding demonstrated that the binding of ICAM-1 expressed on pre-OC surfaces to LFA-1 promotes pre-OC recruitment and adhesion [55]. Other studies have found that the LFA-1/ICAM-1 axis not only exerts promoting effects but may also indirectly inhibit OC formation. Li et al. investigated the effects of skeletal stem cells (SSCs) on OC formation using a lipopolysaccharide-induced mouse model of osteolysis in vivo and human OA synovial fluid (OASF) in vitro. The results showed that the OASF stimulated the expression of ICAM-1 and the secretion of osteoprotegerin (OPG) from the SSCs. OPG is a soluble RANKL receptor, mainly produced by OBs, that prevents OC formation and bone resorption through the inhibition of RANK/RANKL signaling. The recruitment of CD11b+OC progenitors by ICAM-1 is close to SSCs, which strengthens the inhibitory effect of SSC-derived OPG on osteoclastogenesis [56,57].

Mac-1, a receptor expressed on pre-OCs, participates in RANK/RANKL signaling-induced OC differentiation. It is regarded as one of the best markers of pre-OCs and may serve a dual role in OA pathogenesis. Mac-1 plays a synergistic role in the differentiation of bone remodeling cells. Multinucleated OCs are derived from Mac-1-positive macrophages in the bone marrow and circulation. Hayashi et al. found that bone marrow macrophages treated with anti-CD11b antibodies led to the inhibition of RANKL-induced increases in the mRNA levels of the nuclear factor of activated T-cell cytoplasmic 1 (NFATc1), thereby affecting OC formation. This suggests that Mac-1 signaling plays an essential role in the early osteoclastogenesis of bone marrow macrophages [58]. Yang et al. reported that CD11b upregulated the levels of spleen tyrosine kinase (Syk), c-Fos, the nuclear factor of activated T cells, NFATc1, and the activity of extracellular-regulated kinase (Erk). Consequently, pre-OCs were differentiated into tartrate-resistant acid phosphatase (TRAP)-positive cells, marking the formation of OCs [59]. ICAM-2 is a Mac-1 counter receptor expressed on pre-OCs, and the binding between Mac-1 and ICAM-2 is a vital step in early OC formation. The activation of the PI3K/Akt signaling pathway by ICAM-2/Mac-1 interactions may play a key role in osteoclastogenesis [60]. This finding provided a new molecular target for OA. On the basis that the promoting effects of CD11b towards the occurrence of OA have been demonstrated by previous research, the use of CD11b as a therapeutic target was investigated in a subsequent study. Research indicated that the blockade of CD11b receptors inhibits the recruitment of peripheral CD11b+ bone marrow monocytes from blood to the inflammatory site, thereby attenuating inflammatory cell infiltration and OC formation [61]. However, other researchers have asserted that CD11b may exert protective effects during OA occurrence. Park-Min et al. found that CD11b inhibited NFATc1 expression by downregulating RANK expression and inducing the recruitment of the transcriptional repressor BCL6 to the NFATc1 gene. These findings confirm the negative regulatory role of CD11b in the early stages of OC differentiation [62]. In an in vivo experiment conducted in a murine model of collagen-induced arthritis (CIA), CD11b−/− mice were found to develop arthritis with an early onset, high incidence rate, and constant high severity compared with that of control C57BL/6 mice. This study showed that CD11b−/− DCs induced much stronger IL-6 production and, hence, T helper 17 (Th17) cell differentiation than wild-type DCs. The treatment of CD11b−/− mice after the establishment of the regulatory T cell (Treg)/Th17 balance by the IL-6 receptor-neutralizing antibody substantially suppressed the induction of Th17 cells and reduced arthritis severity [63].

In addition to monocytes, CD11c+ cells also serve an important role in osteoclastogenesis. CD11c is a specific surface marker of DCs, with the latter regarded as pre-OC by several researchers [64,65]. The generation of DCs, which differentiate into OCs, is induced through the stimulation of bone marrow-derived osteoclast progenitor cells (OPCs) by the granulocyte–macrophage colony-stimulating factor (GM-CSF) rather than M-CSF or RANKL [66]. Tsukasaki et al. performed a transcriptional analysis of 7228 murine cells undergoing in vitro osteoclastogenesis and found that pre-OCs transiently expressed CD11c. In addition, the specific deletion of RANK expression in CD11c-expressing precursors inhibited OC formation in vivo and in vitro. This suggests that CD11c may exert effects on OC formation through RANK/RANKL signaling [67]. However, the specific mechanisms of action of CD11c molecules in osteoclast formation remain unclear.

### 3.2. Effects of β2 Integrins on Macrophages

Macrophages are effector cells of the innate immune system and play a vital role in maintaining homeostasis in the immune microenvironment [68]. They can be classified as M1 and M2 subtypes based on phenotypic differences in macrophage differentiation after activation. M1 macrophages are regarded as pro-inflammatory cells as they produce inflammatory cytokines (e.g., IL-1β, IL-6, IL-12, IL-23, and TNF-α) upon induction by Toll-like receptors (TLRs). In contrast, M2 macrophages are generally regarded as anti-inflammatory cells which promote tissue repair [69]. In recent years, an increasing number of studies have discovered the important role served by macrophage polarization in the occurrence of OA. For instance, Liu et al. found that the number of macrophages and the M1/M2 macrophage proportion were higher in the synovial membrane of OA patients compared with healthy control subjects. Xie et al. performed flow cytometry and semi-quantitative immunofluorescence analysis on the synovial tissues of OA and found that M1-polarized macrophages were dominant in OA patients [70,71]. Other studies have reported that the inhibition of M1 macrophages and the promotion of M2 polarization to regulate the equilibrium of the immune microenvironment in the joints of OA patients are promising targets for the treatment of OA [72,73].

Protein adsorption and integrin-binding interactions may affect inflammation by affecting macrophage polarization. Lv et al. used small interfering RNA (siRNA) for the treatment of the monocyte/macrophage-like cell line RAW 264.7 with different surface hydrophilicity to specifically inhibit β2 integrin expression. The results indicated that interactions between β2 integrins and hydrophobic surfaces induced the activation of the nuclear factor-kappa B (NF-κB) pathway, leading to M1 macrophage polarization. Therefore, it can be deduced that β2 integrins participate in the induction of NF-κB activation, which promotes the expression of inflammatory cytokines and consequently results in pro-inflammatory macrophage polarization [74]. Although both LFA-1 and Mac-1 play a role in transendothelial migration across the endothelium, an in vitro study found that Mac-1, but not LFA-1, mediates the β2 integrin-dependent migration of PMNL through the fibroblast barriers [75]. As mentioned above, Mac-1 is known as a specific marker expressed on the surfaces of macrophages. This is consistent with existing studies that macrophages are the main immune cells that are present in OA synovial lining. In subsequent studies by Eyles et al., the Mac-1-mediated trafficking of leukocytes into synovial tissue was regarded as a characteristic of arthritis. It was also found that the blockade of Mac-1 attenuated the progression of CIA in wild-type mice, suggesting the possibility that Mac-1 may be utilized as a target for arthritis treatment [76].

### 3.3. Effects of β2 Integrins on Chondrocytes

Cartilage is an important structural component of joints and is mainly composed of chondrocytes. The denaturation and destruction of articular cartilage is a key part of the pathological changes in OA. Cartilage ECM components such as fibronectin, collagen, and laminin are known ligands of integrins. Previous research has indicated that integrins serve a crucial role in the differentiation and survival of chondrocytes [77,78]. A lack of integrin signaling may trigger the catabolic reactions of cartilage matrix degeneration, ultimately resulting in OA [79]. Ehirchiou et al. recently found that CD11b is also expressed in chondrocytes. In a murine model of OA, it was observed that CD11b-deficient mice exhibited increased cartilage degradation compared to the wild-type control mice. The results indicated that CD11b deficiency in chondrocytes enhanced chondrocyte calcification via increased IL-6 production and a switch toward hypertrophic cell differentiation, thereby disrupting articular cartilage homeostasis and overall leading to cartilage degradation. This shows that the CD11b-dependent signaling pathway may exert a protective effect on OA through involvement in the cartilage calcification process [80]. However, the functional significance of the CD11b-dependent signaling pathway in the pathophysiology of chondrocytes remains unclear.

### 3.4. Effects of β2 Integrins on FLS

The occurrence of synovitis plays an important role in pain symptoms and disease progression in OA [81,82]. Pathological changes in synovitis are mainly manifested as synoviocyte hypertrophy and hyperplasia, synovial fibrosis, and leukocyte infiltration. The vast bulk of the hyperplastic synovial membrane is formed by FLS, and its phenotypic changes and interactions with cells form an essential basis for the pathological changes in the synovial membrane [83]. The driving effects of the mutual adhesion of β2 integrins and FLS in RA have already been adequately demonstrated [84,85,86,87]. Adhesion between β2 integrins expressed on leukocyte surfaces and associated ligands expressed on FLS is of vital importance to leukocyte activation and migration [40,88]. Macrophage inflammatory protein-1α (MIP-1α) is a key chemokine that prompts the entry of macrophages and certain T lymphocytes into the articular cavity. Activated leukocytes may synergistically increase MIP-1α expression and secretion through interactions between the β2 integrin/ICAM-1 mechanism and FLSs [87], which is indicative of the relationship between β2 integrins and inflammatory cell transport.

Although the behavior of synoviocytes differs between OA and RA, β2 integrins also participate in the pathophysiology of synovitis in OA. ICAM-1, an important ligand of β2 integrins expressed on FLS surfaces, is generally regarded as a marker of synovitis in OA. Many recent studies have focused on reducing ICAM-1 expression on the surfaces of FLS in animal models of OA in the quest for drugs to alleviate OA synovitis [89,90]. During the occurrence of OA synovitis, there is an abundant influx of leukocytes from blood vessels in response to cytokines and cell adhesion molecules, including the important pro-inflammatory mediator ICAM-1 produced by FLS [91]. This indicates that leukocyte migration also exists in OA synovitis. Macrophage infiltration of the synovial membrane is common in both OA and RA, and β2 integrins are expressed on the surfaces of macrophage-like synovial cells. As previously stated, CD11c (+) CD11b (+) macrophages have been identified as a common inflammatory subgroup in the synovial tissue of OA mice [13]. Leukocyte adhesion and migration mediated by LFA-1/ICAM-1 interaction not only participate in the amplification of the inflammatory response, but are also regarded as the initiating step of angiogenesis and an accelerant of cartilage deformation and destruction [92,93]. These findings suggest that FLS may interact with β2 integrins on macrophage surfaces and mediate leukocyte migration through the production of pro-inflammatory mediators. However, further validation is required.

## 4. The Potential of β2 Integrin Antagonists Serving as a Therapeutic Target of OA

Presently, important progress has been made in the utilization of integrin antagonists for the treatment of inflammatory bowel disease (IBD) [94]. Some drugs including anti-α4β7 integrin antibodies (vedolizumab, abrilumab, etrolizumab) and small molecules (PTG-100, AJM300) have already been researched pre-clinically or clinically [95,96,97,98,99]. However, it is noteworthy that efalizumab, a recombinant monoclonal antibody against the αL-integrin chain, has previously been approved for the treatment of moderate-to-severe psoriasis. Unfortunately, it was then withdrawn from the market due to the appearance of severe adverse events of progressive multifocal leukoencephalopathy in patients on long-term (>3 years) efalizumab therapy [100]. Therefore, safety and effectivity are of paramount importance for drug fabrication by targeting β2 integrins, since these molecules play multifaceted roles in different organs and tissues. These issues must be handled primarily. Firstly, almost all integrin antagonists target either the ligand-binding site or the ligand itself to work, and the specificity of drugs is essential for recognizing the different integrin subunits such as Mac-1 and LFA-1. Moreover, integrins shift between inactive and active conformation. New inhibitory approaches, such as small-molecule integrin blockers for β2 integrin conformational change, may be promising. Furthermore, the strategies of drug delivery and drug dosage are deeply relevant to safety and effectiveness. Considering the pivotal role of β2 integrins in leukocyte recruitment, circumventing the severe adverse events in general health is incredibly important. Meanwhile, post-marketing surveillance is also essential for the correct evaluation of drug potential. Briefly, β2 integrin antagonists with high effectivity, selectivity, and safety, allowing local injection, will lead to a brand-new treatment for OA in the future.

## 5. Conclusions

**Figure 3:**
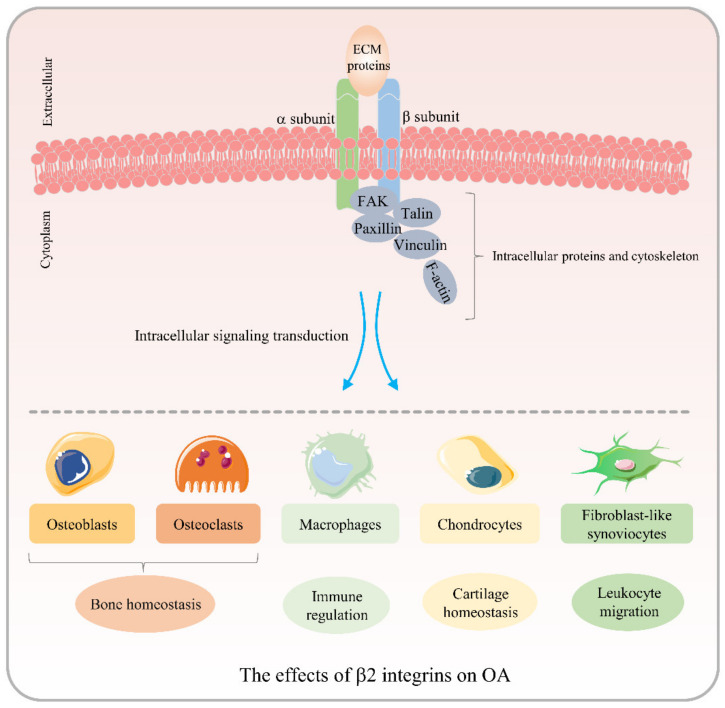
Schematic representation of the roles of β2 integrins in osteoarthritis (OA) pathogenesis. β2 integrins interact with ligands on osteoblast surfaces and may serve a dual role in the differentiation and maturation of osteoclasts, thereby upsetting bone homeostasis in OA. β2 integrins may participate in the induction of NF-κB activation and promote the expression of inflammatory cytokines, consequently resulting in pro-inflammatory macrophage polarization. Mac-1-mediated migration of macrophages to synovial tissue is regarded as a characteristic of arthritis. β2 integrins may maintain cartilage homeostasis by inhibiting chondrocyte calcification. The interactions of β2 integrins with ICAM-1 on fibroblast-like synoviocyte surfaces mediate leukocyte migration to the synovial membrane, thereby inducing the amplification of the inflammatory response, angiogenesis, and acceleration of cartilage injury.

The upregulation of β2 integrin expression in the pathological process of OA has already been well demonstrated. With the in-depth investigation of the roles of β2 integrins as adhesion molecules in the occurrence of OA, β2 integrins possibly become potential targets for OA treatment. Although the exact significance of these findings remains to be elucidated, the role of β2 integrins in bone homeostasis dysregulation, macrophage polarization, the inhibition of cartilage mineralization, and the mediation of leukocyte influx into the synovial membrane indicate that they are key molecules in the pathogenesis of OA (Figure 3). Future research on the mechanisms of the β2 integrin-mediated regulation of inflammation not only offers promise in the elucidation of disease pathogenesis, but may also lead to the discovery of novel methods for the treatment of OA.

## Data Availability

Not applicable.
